# Clustering of loci controlling species differences in male chemical bouquets of sympatric *Heliconius* butterflies

**DOI:** 10.1002/ece3.6947

**Published:** 2020-12-16

**Authors:** Kelsey J. R. P. Byers, Kathy Darragh, Sylvia Fernanda Garza, Diana Abondano Almeida, Ian A. Warren, Pasi M. A. Rastas, Richard M. Merrill, Stefan Schulz, W. Owen McMillan, Chris D. Jiggins

**Affiliations:** ^1^ Department of Zoology University of Cambridge Cambridge UK; ^2^ Smithsonian Tropical Research Institute Panama Panama; ^3^ Institute of Biotechnology University of Helsinki Helsinki Finland; ^4^ Division of Evolutionary Biology Ludwig‐Maximilians‐Universität München Munich Germany; ^5^ Institute of Organic Chemistry Department of Life Sciences Technische Universität Braunschweig Braunschweig Germany; ^6^ Present address: Department of Cell and Developmental Biology John Innes Centre Norwich UK; ^7^ Present address: Department of Evolution and Ecology University of California Davis Davis CA USA; ^8^ Present address: Department of Collective Behaviour Max Planck Institute of Animal Behaviour Konstanz Germany; ^9^ Present address: Institute for Ecology, Evolution and Diversity Goethe Universität Frankfurt Germany

**Keywords:** *Heliconius*, Lepidoptera, pheromones, quantitative trait locus mapping, speciation

## Abstract

The degree to which loci promoting reproductive isolation cluster in the genome—that is, the genetic architecture of reproductive isolation—can influence the tempo and mode of speciation. Tight linkage between these loci can facilitate speciation in the face of gene flow. Pheromones play a role in reproductive isolation in many Lepidoptera species, and the role of endogenously produced compounds as secondary metabolites decreases the likelihood of pleiotropy associated with many barrier loci. *Heliconius* butterflies use male sex pheromones to both court females (aphrodisiac wing pheromones) and ward off male courtship (male‐transferred antiaphrodisiac genital pheromones), and it is likely that these compounds play a role in reproductive isolation between *Heliconius* species. Using a set of backcross hybrids between *H. melpomene* and *H. cydno*, we investigated the genetic architecture of putative male pheromone compound production. We found a set of 40 significant quantitative trait loci (QTL) representing 33 potential pheromone compounds. QTL clustered significantly on two chromosomes, chromosome 8 for genital compounds and chromosome 20 for wing compounds, and chromosome 20 was enriched for potential pheromone biosynthesis genes. There was minimal overlap between pheromone QTL and known QTL for mate choice and color pattern. Nonetheless, we did detect linkage between a QTL for wing androconial area and *optix*, a color pattern locus known to play a role in reproductive isolation in these species. This tight clustering of putative pheromone loci might contribute to coincident reproductive isolating barriers, facilitating speciation despite ongoing gene flow.

## Graphical abstract

The degree to which loci promoting reproductive isolation cluster in the genome—that is, the genetic architecture of reproductive isolation—can influence the tempo and mode of speciation. We found that QTL for wing and genital pheromone production in Heliconius butterflies, which might play a role in reproductive isolation, clustered significantly on two chromosomes, chromosome 8 for genital compounds and chromosome 20 for wing compounds, and chromosome 20 was enriched for potential pheromone biosynthesis genes. This tight clustering of putative pheromone loci might contribute to coincident reproductive isolating barriers, facilitating speciation despite ongoing gene flow.

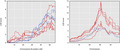

## 1 INTRODUCTION

The genetic architecture of population differences can profoundly affect the evolution and maintenance of new species, especially in the face of ongoing gene flow. For example, large effect loci are expected to facilitate speciation (Merrill et al., 2019; Via, 2012) because they are less likely to be lost to drift (Kimura, 1983) and a single large effect locus can lead to substantial reproductive isolation on its own (Bradshaw & Schemske, 2003). Similarly, tight physical linkage of barrier loci, that is, those that contribute to reproductive isolation, will promote speciation by impeding the breakdown of genetic associations between traits that isolate emerging species (Felsenstein, 1981; Smadja & Butlin, 2011). As a result, there is considerable interest in finding the genetic basis of traits that contribute to reproductive isolation and understanding both the effect size and distribution of loci across the genome.

Physical linkage between barrier traits has been detected in a variety of taxa, including pea aphids (Hawthorne & Via, 2001), *Laupala* crickets (Wiley et al., 2011), *Heliconius* butterflies (Merrill et al., 2019), *Ficedula* flycatchers (Sæther et al., 2007), stickleback fish (Bay et al., 2017), and *Aquilegia* columbines (Hodges et al., 2002). Barrier traits can include sex pheromones, chemical signals that mediate intraspecific communication important for mating. Due to their critical role in mate attraction, and their ability to convey information about species identity and male quality, pheromones can be important for establishing and maintaining reproductive isolation through relatively simple changes in chemical bouquets (Smadja & Butlin, 2009). As products of secondary metabolism (*i.e.,* products not required for survival), alterations in endogenously produced or modified pheromones also have the potential to be both relatively simple at the molecular level and, if such changes are relatively late in the pathway, likely avoid major pleiotropic consequences.

Some of the best studied sex pheromones involved in speciation are those of female Lepidoptera, and in some cases, the genetic basis of variation in these pheromones is now well known. Both desaturases and fatty acyl‐CoA reductases appear to be commonly involved in pheromone biosynthetic variation. Desaturases introduce double or triple bonds between carbon molecules in pheromone components, for example turning alkanes (less common as pheromone components) into alkenes (a common component of lepidopteran pheromones). Fatty acyl‐CoA reductases, or FARs, turn fatty acids into fatty alcohols, for example turning octadecanoic acid into octadecanol. In *Ctenopseustis*, for example, differential regulation of a desaturase drives differences in ratios of sex pheromone components between species (Albre et al., 2012). Desaturases are also important in *Ostrinia* (Fujii et al., 2015; Roelofs et al., 2002; Sakai et al., 2009) and *Helicoverpa* (Li et al., 2015, 2017). Fatty acyl‐CoA reductases are responsible for species‐specific pheromone alterations in *Ostrinia nubialis* strains (Lassance et al., 2010, 2013). Within single species, desaturases and FARs have received the most attention, being functionally characterized in a variety of Lepidoptera including *Yponomeuta* (Liénard et al., 2010), *Agrotis* (Ding & Löfstedt, 2015), *Manduca* (Buček et al., 2015), *Bombyx* (Moto et al., 2004), and *Antheraea* (Wang et al., 2010). In some cases, variation in these female pheromones has also been shown to contribute to reproductive isolation (e.g., Liebherr & Roelofs, 1975, Wu et al., 1999, Emelianov et al., 2001; reviewed in Smadja & Butlin, 2009).

In contrast, male pheromones in Lepidoptera appear more varied, with a diverse array of compound classes (Conner & Iyengar, 2016; Löfstedt et al., 2016). Many male Lepidopteran pheromones resemble plant compounds and are thought to be diet‐derived, especially terpenoids and pyrrolizine alkaloids, the latter of which can be derived from either larval or adult feeding (Conner & Iyengar, 2016). In comparison with female pheromones, the genetic basis of variation in male sex pheromones has received less attention. In male *Bicyclus anynana*, a desaturase (*Ban‐Δ11*) and two FARs (*Ban‐wFAR1* and *Ban‐wFAR2*) produce pheromone components and precursors (Liénard et al., 2014), and in the moth *Ostrinia nubialis*, males use the same Δ11‐desaturase and Δ14‐desaturase as females to produce pheromones (Lassance & Löfstedt, 2009). However, the role of these genes in mediating differences between species remains unclear.

Speciation is typically thought to rely on the accumulation of multiple reproductive barriers, involving divergence in many different traits (Butlin & Smadja, 2017). In order to understand the extent of genetic linkage that underlies species differences, it will therefore be useful to study taxa in which multiple different traits can be mapped. *Heliconius* butterflies have been well studied in the context of speciation (Bates, 1861; Jiggins, 2017; Merrill et al., 2015), and in particular, we know a great deal about the species complex of *Heliconius melpomene* and its sister lineage *Heliconius cydno/timareta*. Genetic loci underlying wing pattern and mate preference have been mapped in this group (Jiggins, 2017; Merrill et al., 2010, 2019). The additional role of chemical signaling in reproductive isolation has long been suspected (Jiggins, 2008), but only recently studied in any detail. Chemical profiles of the male wing androconia (patches of specialist scales on male wings that release pheromones) and genital regions (thought to act as aphrodisiacs and antiaphrodisiacs, respectively) differ between species (Darragh et al., 2020; Estrada et al., 2011; Mann et al., 2017) and are important for mate choice, including altering behavior toward con‐ and heterospecific individuals (Darragh et al., 2017; González‐Rojas et al., 2020; Mérot et al., 2015). Genital pheromones play a more complex role in mate choice in *Heliconius*, as they are transferred by males to females during mating and subsequently decrease advances by other males (Estrada, 2009; Gilbert, 1976; Schulz et al., 2007).

**FIGURE 1:**
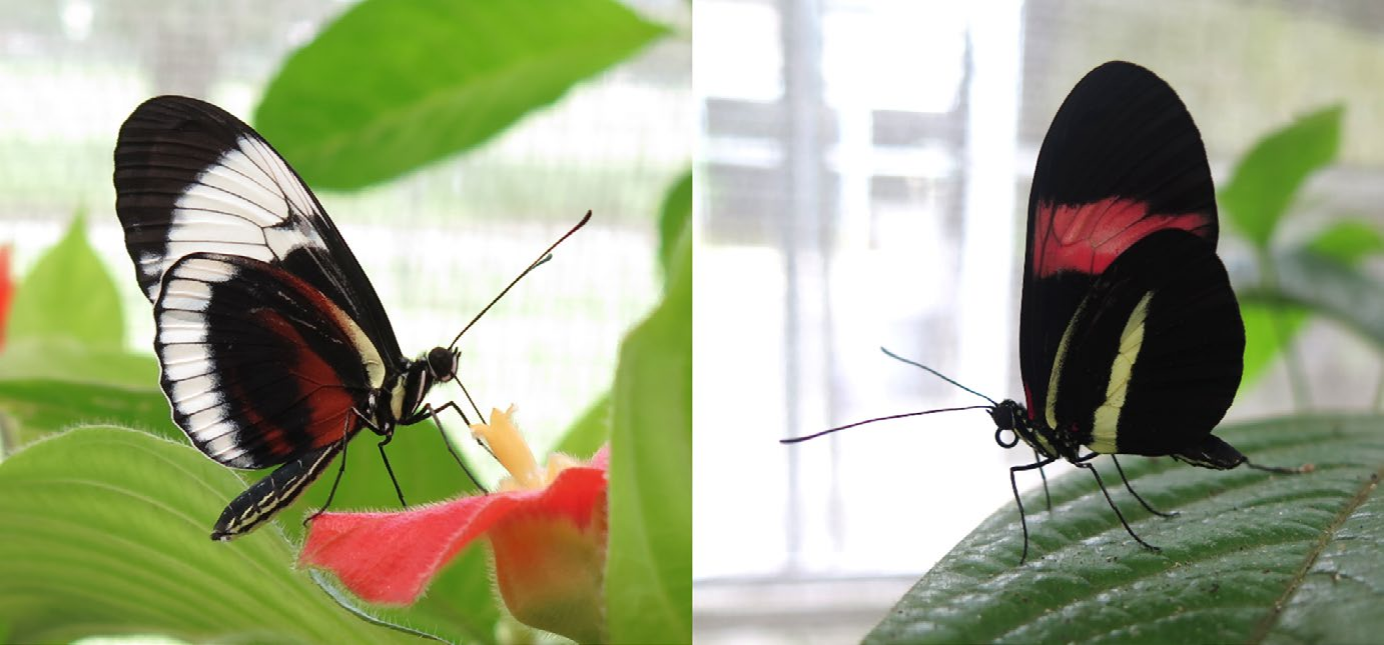
*Heliconius cydno chioneus*(left) and*Heliconius melpomene rosina*(right), the two butterfly species whose putative pheromone components and wing androconial area are mapped in this study

One challenge of studying the entire chemical profile is determining which combination of compounds are behaviorally active. The chemical bouquets of wings and genitals are complex, often consisting of 30‐70 compounds (Byers et al., 2020; Darragh et al., 2017, 2020; Mann et al., 2017, this study), but it remains unknown how many of these are important for signaling. A single wing compound in *H. melpomene*, octadecanal, is known to be biologically active in *H. melpomene* and *H. cydno* (Byers et al., 2020), while the genital compound (*E*)‐β‐ocimene acts as the main antiaphrodisiac in *H. melpomene* (Schulz et al., 2007). In addition, one bioactive genital compound in *H. cydno* (hexyl 3‐methylbutyrate, Estrada, 2009) is known. It seems likely that additional compounds in the complex bouquet (30–70 compounds) produced by each of these species are biologically active, but this remains to be tested. The simple genetic control of pheromone components in other systems and their role as secondary metabolism products suggests that they can be altered relatively simply without major pleiotropic consequences to essential organismal functions, as has been seen in some moth species (Groot et al., 2019), though pleiotropic effects have been seen in *Drosophila* (Bousquet et al., 2012; Zelle et al., 2020). In *Drosophila,* some loci specifically showed pleiotropy of production and perception of pheromones, thought to be rare in Lepidoptera (Haynes, 2016). In combination with their importance in inter‐ and intraspecific mate choice, such simple genetic control and relatively lower risk of pleiotropy makes them ripe material for adaptation and speciation. We here conduct quantitative trait locus (QTL) analyses for wing androconial area and wing and genital compounds that differ between *Heliconius melpomene* L. and *H. cydno* Doubleday (Figure 1; see also Byers et al., 2020, Darragh et al., 2020). We then investigated the patterns of QTL distribution across the genome to test for clustering of loci across chromosomes.

## 2 METHODS

*Heliconius melpomene rosina* and *H. cydno chioneus* (hereafter *H. melpomene* and *H. cydno*) and their interspecific hybrids were reared in outdoor insectaries at the Smithsonian Tropical Research Institute in Gamboa, Panama. Initial stocks were established from wild‐caught individuals, and outbred stocks were refreshed with more wild‐caught individuals as necessary. Sexes were kept separately, and virgin females were kept separate from egg‐laying females. To control for host plant effects, all larvae were fed on the same host plant (*Passiflora platyloba* var. *williamsi*), and adults were maintained on a 20% sugar solution and *Gurania eriantha*, *Psiguria triphylla*, *Psiguria warscewiczii*, and *Psychotria poeppigiana* as pollen sources. Pheromones were collected from male individuals (both parental species and offspring of mapping crosses) following (Darragh et al., 2017). Wing and genital data from *H. melpomene* and wing data from *H. cydno* have previously been published in Darragh et al. (2017), Darragh, et al. (2019a), and Byers et al. (2020). Briefly, males of 7–14 days posteclosion had their wings and genitals removed and soaked in dichloromethane plus 1 ng/μL 2‐tetradecyl acetate (DCM + IS) for one hour, after which the DCM + IS was removed to a fresh vial and stored at −20°C before gas chromatography–mass spectrometry analysis. Bodies were collected and preserved in 20% dimethyl sulfoxide (DMSO) and 0.25 M EDTA (pH 8.0) and stored at −20°C for later DNA extraction.

Wing and genital extractions were run on an Agilent 7890B gas chromatograph coupled with an Agilent 5977 mass spectrometer (GC‐MS) with electron ionization (Agilent Technologies, California, USA) with an HP‐5MS capillary column (30 m length, 0.25 mm inner diameter) and helium as the carrier gas (1.2 mL/min). Injection used an Agilent ALS 7693 autosampler and was splitless, with an inlet temperature of 250°C. The oven was first held isothermally at 50°C for five minutes, then increased at 5°C/min to 320°C and again held isothermally for five minutes. Compounds were identified with a custom MS library and quantified using the internal standard area. Only compounds that could be definitively identified were included in the data set. To be included in the mapping experiment, compounds had to be present in at least two thirds of the individuals from one of the parent species, that is, they had to be “typical” *H. melpomene* or *H. cydno* compounds. A limited set of wing compounds (three methyloctadecanals and two henicosenes from *H. melpomene* and *H. cydno,* respectively) were excluded from the wing analysis due to close retention times in the GC‐MS data preventing definitive identification of the species phenotype in the mapping individuals. Absolute androconial area and absolute hindwing area were measured in the GNU Image Manipulation Program (GIMP) as pixel counts, then absolute androconial area was divided by hindwing area to produce the percentage of the hindwing taken by the androconial region, the relative androconial area.

Mapping crosses consisted of backcrosses of either an *H. cydno* or *H. melpomene* mother and an F_1_ father, as F_1_ females are normally sterile. Ten families were constructed as backcrosses to *H. melpomene* (representing 89 individuals for the wing phenotype, 81 individuals for the genital phenotype, and 78 individuals for the hindwing relative androconial area phenotype), and fifteen families were constructed as backcrosses to *H. cydno* (127 individuals for the wing, 114 individuals for the genital phenotype, and 124 individuals for the hindwing relative androconial area phenotype) for a total of 216 and 195 hybrid individuals for wing and genital studies, respectively. DNA extraction, library preparation, and QTL map construction are detailed in Byers et al. (2020) and Darragh, et al., bioRxiv. Briefly, Qiagen DNeasy kits (Qiagen) were used for DNA extraction, and individuals then genotyped either by RAD‐seq or low‐coverage whole genome sequencing using nextera‐based libraries (Davey et al., 2017; Merrill et al., 2019; Picelli et al., 2014). Samples were sequenced by HiSeq 3000 (Illumina) by BGI (China). Linkage mapping was conducted using the standard Lep‐MAP3 (LM3) pipeline (Rastas, 2017). The initial linkage groups and marker order were constructed based on the *H. melpomene* genome for 21 chromosomes, as *H. melpomene* and *H. cydno* have highly colinear genomes (Davey et al., 2017). This resulted in a linkage map with 447,818 SNP markers, which was then evenly thinned by a factor of ten to 44,782 markers to ease computation.

To determine which cross direction to use for each compound, we inspected the distribution of the compound phenotype in the parents, F_1_ individuals, and the backcross individuals. Compounds that were present in equal amounts in both parent species (assessed using a Kruskal–Wallis test) were not mapped. Once cross direction was determined, the remaining compounds were log‐transformed to approximate normality before being regressed against the linkage map using R/qtl2 (Broman et al., 2018). Due to the family structure present in our crosses, we additionally included a kinship matrix calculated by R/qtl2 using the LOCO (leave one chromosome out) method. Permutation testing was used (with 1000 replicates) to determine QTL threshold. Compounds are likely to be correlated, and our data set represents multiple testing of the same mapping crosses; therefore, we used Bonferroni correction separately for each cross direction to establish a second, more conservative, LOD threshold for significance by dividing the threshold by the number of compounds (traits) mapped in the relevant backcross direction. No correction was used for relative androconial area mapping as the trait was mapped alone due to its general lack of correlation with individual volatile compounds. QTL confidence intervals were obtained using bayes_int. As R/qtl2 does not provide a function to calculate the traditional percentage of variance explained by a specific marker, we instead calculated the percentage of the parental difference explained by the genotype at a given marker as a fraction with the difference between the average phenotype of the two genotypes as the numerator and the difference between the average phenotype of the two parental species as the denominator. This can result in values over 100% when more variance is present in the mapping population than the difference between the parent species accounts for.

To identify clustering of QTL, we first calculated the expected number of QTL per chromosome, taking into account the chromosome’s length in cM, then compared that against the observed distribution of QTL per chromosome using a chi‐squared test, computing p‐values with Monte Carlo permutation with B = 10,000 permutations. Examination of the residuals was used to determine which chromosome(s) displayed clustering, with any residual over 2 considered significant, as in Erickson et al. (2016). We also repeated these tests taking into account chromosome length in basepairs because the relationship between map length and physical length can differ across chromosomes. The results were unchanged.

As our linkage map is based on whole genome sequencing data, we were able to recover the basepair intervals corresponding to the QTL centimorgan (cM) confidence intervals (defined as the outermost markers at those cM positions) as well as their peaks (defined as the first marker in at that cM position). Lepbase (Challis et al., 2016) was queried to identify genes within the main interval on chromosome 20 for wing fatty acid‐derived compound production (identified as the minimum overlapping window of all Bonferroni‐significant compound confidence intervals). Genes were searched against the nr (nonredundant) protein database using BLASTp (Altschul et al., 1990) to obtain putative functional annotations, with no specific cutoff used to define a putative annotation; annotations were made when multiple hits with high alignment scores agreed on putative function.

## 3 RESULTS

**FIGURE 2:**
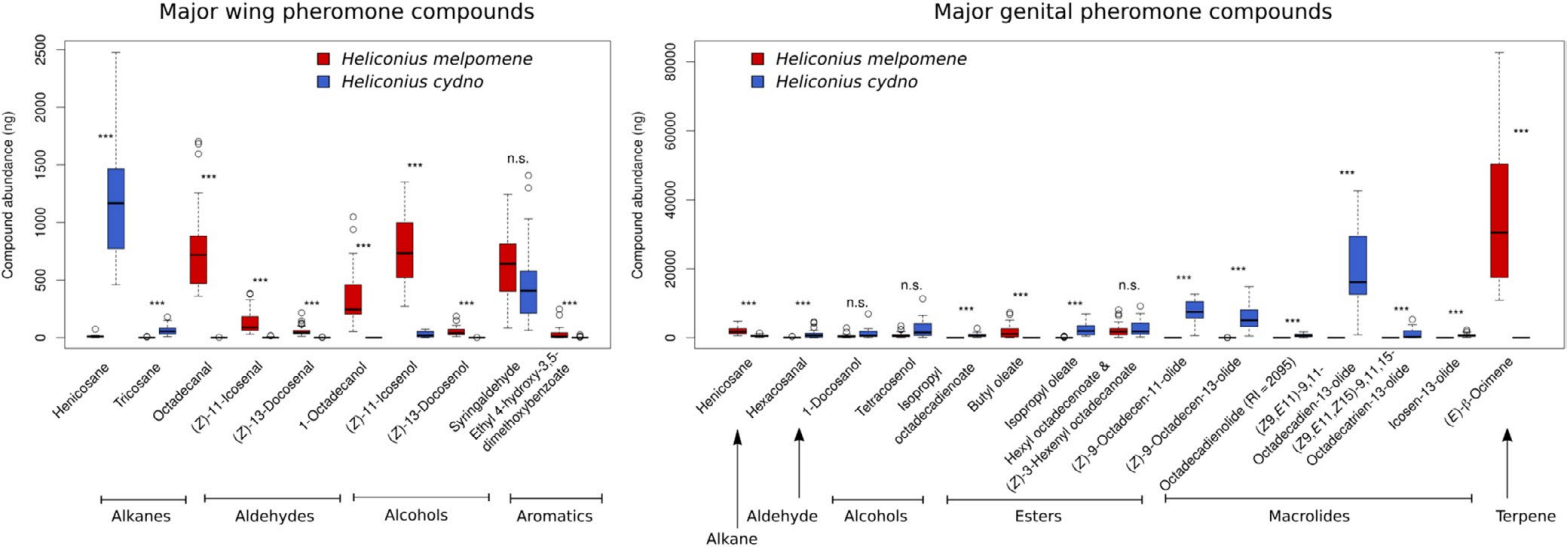
Major compounds found in the wings (left) and genitals (right) of*Heliconius melpomene*and*H. cydno*, arranged by compound type. Only those compounds comprising at least 1% of either species’ pheromone bouquet are included. n.s., not significant; ***:*p* < .005. Boxplots: line is the median, box outline the first and third quartiles, whiskers the most extreme point no more than 1.5 times the interquartile range. [image rotated for reviewer ease of viewing]

We first looked at the distribution of compounds in *H. melpomene*, *H. cydno*, and their hybrids. Analysis of the pheromones found a total of 31 compounds in the wings and 68 compounds in the genitals across the two species that were possible to map (i.e., not unknown or overlapping in the gas chromatography–mass spectrometry trace) (Figure 2, Table 1). There was limited overlap in compounds between the wings and genitals, with only the straight‐chain alkanes heneicosane, docosane, tricosane, pentacosane, and hexacosane (all of which may be part of the normal whole‐body cuticular hydrocarbon profile), the unsaturated aldehyde (*Z*)‐11‐icosenal, and the nitrogenous aromatic benzyl cyanide found in both body regions. A subset of compounds (6 in the wings and 18 in the genitals) were not mapped as they did not differ significantly between the parental species. The rest showed significant differences and thus had adequate variation for QTL mapping to be feasible. Of the wing compounds, 11 showed segregation in backcrosses to *H. cydno* and thus were mapped with those individuals; nine were mapped in backcrosses to *H. melpomene*; and five showed segregation in both backcross directions and thus were mapped separately in backcrosses to each species. For the genital compounds, 19 were mapped in backcrosses to *H. cydno*; 17 in backcrosses to *H. melpomene*; and 14 in both directions.

**TABLE 1:** Full list of compounds found in the wings and genitalia of *Heliconius melpomene* and *H. cydno*, details of statistical comparisons of amounts in both species, and cross direction(s) the compound was mapped in, if any. Coefficient of variance in backcrosses is the variance within the backcross direction divided by the mean amount found within that backcross direction, to control for variance differences due to compound abundance differences

Compound	Compound type	Mean *H. cydno*	Mean *H. melpomene*	Kruskal–Wallis chi‐squared statistic	Bonferroni‐adjusted *p*‐value	Coefficient of variance in backcrosses to *H. melpomene*	Coefficient of variance in backcrosses to *H. cydno*	Mapped in backcrosses to
Wings								
Icosane	Alkane	6.977	0.033	41.397	3.85E‐09	0.808	2.015	*H. melpomene*
Henicosane	Alkane	1152.641	12.447	38.667	1.56E‐08	0.564	1.158	Both species
Docosane	Alkane	13.548	0.012	42.335	2.38E‐09	0.961	2.208	*H. cydno*
Tricosane	Alkane	62.532	1.146	38.938	1.36E‐08	0.973	1.130	*H. cydno*
Pentacosane	Alkane	4.568	8.458	3.732	1.00000	1.197	1.385	Not tested (no species difference)
Hexacosane	Alkane	0.633	8.09	17.348	0.00096	2.081	1.943	*H. melpomene*
Octadecanol	Saturated alcohol	Absent	345.636	42.618	2.06E‐09	2.700	1.310	Both species
Methyloctadecanol	Saturated alcohol	Absent	12.232	42.618	2.06E‐09	5.689	1.028	*H. melpomene*
Octadecanal	Saturated aldehyde	0.569	783.420	39.560	9.86E‐09	1.766	0.909	both species
Nonadecanal, methyl branched	Saturated aldehyde	Absent	4.504	24.238	2.64E‐05	11.269	1.739	*H. melpomene*
Icosanal	Saturated aldehyde	0.432	22.596	40.297	6.76E‐09	2.064	0.630	*H. cydno*
Tricosene (RI = 2265)	Alkene	3.112	Absent	26.042	1.04E‐05	1.952	3.985	*H. cydno*
Tricosene (RI = 2276)	Alkene	0.405	7.215	35.296	8.78E‐08	4.060	1.729	Both species
(*Z*)‐16‐methyl‐9‐octadecenol	Unsaturated alcohol	Absent	7.501	40.267	6.87E‐09	11.269	1.216	*H. melpomene*
(*Z*)‐11‐icosenol	Unsaturated alcohol	28.174	772.157	38.667	1.56E‐08	1.645	0.614	*H. cydno*
(*Z*)‐13‐docosenol	Unsaturated alcohol	0.037	54.766	42.102	2.69E‐09	4.636	1.938	both species
(*Z*)‐9‐octadecenal	Unsaturated aldehyde	Absent	10.938	40.267	6.87E‐09	5.378	1.284	*H. melpomene*
(*Z*)‐11‐icosenal	Unsaturated aldehyde	3.29	150.687	39.756	8.92E‐09	1.718	0.748	*H. cydno*
(*Z*)‐13‐docosenal	Unsaturated aldehyde	0.276	62.556	41.226	4.20E‐09	1.958	0.865	*H. cydno*
Naphthalene	Aromatic	0.752	1.690	9.981	0.04903	1.666	1.731	*H. melpomene*
Syringaldehyde	Aromatic	463.585	644.064	3.724	1.00000	0.656	0.567	Not tested (no species difference)
3,5‐Dimethoxy‐4‐hydroxy benzyl alcohol	Aromatic	3.58	4.162	0.373	1.00000	1.386	1.500	Not tested (no species difference)
Methyl salicylate	Aromatic	2.848	3.348	0.347	1.00000	0.967	1.300	Not tested (no species difference)
Ethyl 4‐hydroxy‐3,5‐dimethoxybenzoate	Aromatic	2.856	35.573	15.736	0.00226	1.594	1.133	*H. cydno*
Ethanone, 1,4‐hydroxy‐3,5‐dimethoxyphenyl‐	Aromatic	1.992	2.981	2.918	1.00000	1.559	1.374	Not tested (no species difference)
Propenone, 1,4‐hydroxy‐3,5‐dimethoxyphenyl‐	Aromatic	2.052	3.932	5.665	0.53653	1.776	1.557	Not tested (no species difference)
Benzyl cyanide	Aromatic (nitrogenous)	1.813	Absent	30.542	1.01E‐06	1.172	2.074	Both species
Methyl 3‐indoleacetate	Aromatic (nitrogenous)	0.013	3.782	32.668	3.39E‐07	2.038	0.923	*H. cydno*
Methyl 3‐indolecarboxylate	Aromatic (nitrogenous)	Absent	2.098	25.994	1.06E‐05	3.205	1.106	*H. melpomene*
*cis*‐Linalool oxide	Terpenoid	3.407	Absent	30.542	1.01E‐06	1.536	9.434	*H. cydno*
Dihydroactinidiolide	Terpenoid	3.020	1.236	11.507	0.02149	0.937	0.567	Both species
Genitals								
Icosane	Alkane	3.115	18.638	22.192	0.0002	1.328	1.355	*H. cydno*
Henicosane	Alkane	550.519	1964.756	30.882	1.87E‐06	0.977	0.771	*H. cydno*
Docosane	Alkane	Absent	28.170	40.267	1.51E‐08	1.630	3.035	Both species
Tricosane	Alkane	45.678	167.126	18.854	0.0010	1.325	1.132	*H. cydno*
Pentacosane	Alkane	8.393	109.178	32.664	7.45E‐07	0.958	1.795	Both species
11‐Methylpentacosane	Alkane	Absent	45.773	29.705	3.42E‐06	2.220	4.709	*H. cydno*
Hexacosane	Alkane	Absent	18.852	24.238	5.79E‐05	3.424	5.194	*H. cydno*
Heptacosane	Alkane	352.867	146.631	6.057	0.9419	1.208	1.683	Not tested (no species difference)
1‐Docosanol	Saturated alcohol	1187.774	516.916	4.296	1.0000	1.786	1.117	Not tested (no species difference)
1‐Tetracosanol	Saturated alcohol	171.351	362.030	5.295	1.0000	1.798	2.115	Not tested (no species difference)
1,3‐Docosanediol (cyclic dimethylsilyl derivative)	Saturated alcohol	297.605	141.388	9.778	0.1201	1.893	1.742	Not tested (no species difference)
1,3‐Tricosanediol (cyclic dimethylsilyl derivative)	Saturated alcohol	195.132	223.311	1.702	1.0000	2.076	1.780	Not tested (no species difference)
Hexacosanal	Saturated aldehyde	1093.627	76.717	19.523	0.0007	1.907	1.728	*H. melpomene*
Hexyl 3‐methylbutyrate	Saturated ester	421.064	Absent	40.612	1.26E‐08	3.050	1.466	Both species
Hexyl hexanoate	Saturated ester	111.474	Absent	43.371	3.08E‐09	NA (absent)	2.054	*H. cydno*
Butyl hexadecanoate	Saturated ester	Absent	257.447	35.810	1.48E‐07	2.706	NA (absent)	*H. melpomene*
Octadecanoic acid ester	Saturated ester	43.400	4.679	18.310	0.0013	2.249	1.498	Both species
Butyl octadecanoate	Saturated ester	Absent	88.671	29.705	3.42E‐06	2.514	NA (absent)	*H. melpomene*
Isopropyl octadecanoate	Saturated ester	97.088	Absent	43.371	3.08E‐09	3.986	2.100	*H. melpomene*
Hexyl octadecanoate (RI = 2550)	Saturated ester	24.217	121.817	11.402	0.0498887	3.289	2.790	Not tested (no species difference)
Hexyl octadecanoate (RI = 2590)	Saturated ester	415.139	0.259	41.397	8.45E‐09	3.158	1.552	*H. melpomene*
Henicosene (RI = 2068)	Alkene	Absent	80.148	33.700	4.37E‐07	1.030	1.589	*H. cydno*
Henicosene (RI = 2072)	Alkene	81.486	30.527	5.041	1.0000	1.214	2.130	Not tested (no species difference)
Tricosene (RI = 2275)	Alkene	217.571	38.420	6.746	0.6391	1.257	1.238	Not tested (no species difference)
Tricosene (RI = 2270)	Alkene	64.338	135.602	10.462	0.0829	1.357	1.246	Not tested (no species difference)
(*Z*)‐13‐Docosen‐1‐ol	Unsaturated alcohol	71.558	102.798	3.592	1.0000	1.200	1.511	Not tested (no species difference)
Tetracosenol	Unsaturated alcohol	2559.806	786.420	10.576	0.0779	1.529	1.456	Not tested (no species difference)
(*Z*)‐3‐Hexenyl isobutyrate	Unsaturated ester	26.306	Absent	28.250	7.25E‐06	3.315	1.828	Both species
Hexyl hexadecenoate	Unsaturated ester	63.084	7.823	20.025	0.0005	2.258	1.997	Both species
Butyl oleate	Unsaturated ester	Absent	1760.544	42.618	4.52E‐09	1.931	4.234	Both species
Isopropyl oleate	Unsaturated ester	2259.264	50.216	40.297	1.48E‐08	2.401	2.288	*H. melpomene*
(*Z*)‐3‐Hexenyl hexadecanoate	Unsaturated ester	97.210	374.827	11.170	0.0565	1.919	1.908	not tested (no species difference)
Isopropyl octadecadienoate	Unsaturated ester	740.075	Absent	46.234	7.13E‐10	3.590	1.590	*H. melpomene*
Isopropyl 9,12‐octadecadienoate	Unsaturated ester	405.054	Absent	46.234	7.13E‐10	4.420	1.695	Both species
Hexyl octadecenoate and (Z)‐3‐Hexenyl octadecenoate	Unsaturated ester	2898.234	2253.323	1.037	1.0000	1.472	1.399	not tested (no species difference)
Hexenyl octadecatrienoate & (*Z*)‐3‐Hexenyl octadecatrienoate	Unsaturated ester	367.972	352.581	0.707	1.0000	1.954	2.049	Not tested (no species difference)
(*Z*)‐3‐Hexenyl octadecanoate	Unsaturated ester	35.961	139.479	10.460	0.0830	2.432	3.251	Not tested (no species difference)
5‐Decanolide	Lactone	36.466	Absent	32.920	6.53E‐07	NA (absent)	2.610	*H. cydno*
15‐Tetradecanolide	Macrolide	41.895	14.383	8.691	0.2175	1.672	1.720	Not tested (no species difference)
Brassicalactone	Macrolide	127.495	Absent	43.371	3.08E‐09	7.229	3.880	*H. cydno*
16‐Hexadecanolide	Macrolide	89.514	0.670	35.448	1.78E‐07	3.725	2.279	*H. cydno*
Hexadecenolide	Macrolide	165.488	6.866	36.495	1.04E‐07	2.022	1.125	*H. melpomene*
Hexadecen‐11‐olide	Macrolide	110.815	Absent	43.371	3.08E‐09	2.821	1.560	Both species
9,11‐Hexadecadien‐11‐olide	Macrolide	72.479	Absent	37.952	4.93E‐08	3.581	3.002	*H. melpomene*
12‐Octadecanolide	Macrolide	105.430	Absent	46.234	7.13E‐10	4.128	1.714	*H. cydno*
18‐Octadecanolide	Macrolide	168.048	7.014	38.587	3.56E‐08	1.781	1.794	Both species
Octadecen‐11‐olide	Macrolide	175.211	Absent	43.371	3.08E‐09	4.519	1.431	Both species
(*E*)‐Octadec‐9‐en‐12‐olide	Macrolide	345.939	Absent	46.234	7.13E‐10	2.973	1.382	*H. melpomene*
(*Z*)‐9‐Octadecen‐11‐olide	Macrolide	7508.774	Absent	46.234	7.13E‐10	2.408	1.018	*H. melpomene*
(*Z*)‐9‐Octadecen‐13‐olide	Macrolide	5855.658	7.282	42.618	4.52E‐09	3.164	1.189	*H. melpomene*
9‐Octadecen‐18‐olide	Macrolide	350.327	8.330	39.121	2.71E‐08	1.990	2.463	*H. cydno*
(*Z*9,*E*11)‐ 9,11‐Octadecadien −13‐olide	Macrolide	19714.248	Absent	46.234	7.13E‐10	3.522	1.569	*H. melpomene*
Octadeca‐9,11‐dien‐13‐olide and 11‐Octadecanolide	Macrolide	77.716	Absent	26.042	2.27E‐05	8.944	2.486	*H. cydno*
Octadecadienolide (RI = 2095)	Macrolide	642.378	Absent	46.234	7.13E‐10	4.887	2.099	*H. cydno*
Octadecadienolide (RI = 2178)	Macrolide	101.588	Absent	30.542	2.22E‐06	5.516	1.763	*H. cydno*
(*Z*9,*E*11,*Z*15)‐9,11,15‐ Octadecatrien‐13‐olide	Macrolide	1052.733	Absent	40.612	1.26E‐08	2.743	1.659	*H. melpomene*
13‐Icosanolide	Macrolide	45.901	Absent	40.612	1.26E‐08	8.011	2.404	*H. cydno*
Icosen‐13‐olide	Macrolide	781.655	Absent	46.234	7.13E‐10	3.350	1.718	*H. melpomene*
Docosen‐22‐olide	Macrolide	109.681	Absent	26.042	2.27E‐05	3.537	2.431	*H. melpomene*
Tetracosenolide	Macrolide	489.085	Absent	43.371	3.08E‐09	4.259	1.714	Both species
Benzyl cyanide	Aromatic (nitrogenous)	287.978	Absent	46.234	7.13E‐10	3.830	1.128	Both species
2‐*sec*‐Butyl‐3‐methoxypyrazine	Aromatic (nitrogenous)	3.569	32.656	29.318	4.18E‐06	0.821	1.846	*H. cydno*
2‐Isobutyl‐3‐methoxypyrazine	Aromatic (nitrogenous)	21.148	13.267	1.094	1.0000	1.043	0.920	Not tested (no species difference)
β‐Myrcene	Terpenoid	Absent	12.799	31.666	1.25E‐06	1.537	4.384	*H. cydno*
(*Z*)‐β‐Ocimene	Terpenoid	0.930	55.408	40.857	1.11E‐08	1.303	7.025	Both species
(*E*)‐β‐Ocimene	Terpenoid	Absent	35192.726	42.618	4.52E‐09	0.812	3.982	*H. cydno*
Alloocimene	Terpenoid	Absent	16.255	27.815	9.08E‐06	1.886	7.272	*H. melpomene*
Dihydroedulan II	Terpenoid	20.336	24.824	0.072	1.0000	0.948	1.003	Not tested (no species difference)

Most compounds mapped did not produce significant QTL at either the *p* = .05 or the Bonferroni‐adjusted cutoff levels (the latter taking into account the number of compounds mapped) (Figures S1 and S2). A total of 21 genital compounds and 14 wing compounds produced significant QTL, including three peaks already published in (Byers et al., 2020, Darragh, et al., bioRxiv) for octadecanal, 1‐octadecanol, and (*E*)‐β‐ocimene. Of these QTL, nine genital and ten wing compounds were significant at the more conservative Bonferroni threshold (Tables 2 and 3; Table 4). The higher drop‐out rate for genital compounds after Bonferroni correction is likely the result of the more stringent correction due to a higher number of compounds corrected for. We also found a single significant QTL on chromosome 18 for relative hindwing androconial area in backcrosses to *H. cydno* (Figure S3). The percentage of parental difference explained by the peak markers at these QTL was highly variable, with values ranging from 0.6% to 130% for wing compounds and 1.9‐170% for genital compounds, but most compounds fell within a range of approximately 20‐50% of parental difference explained by the locus. Surprisingly, there was no obvious association between the amount of variance present in the backcrosses and the presence or absence of a QTL for each compound, as seen from the coefficient of variance values in Table 1, which did not differ between compounds that did and did not have a QTL‐associated (*t* = 0.342, df = 78, *p* = .73).

**TABLE 2:** Details of compounds with Bonferroni‐significant QTL in *Heliconius* wing androconia. a: from (Byers et al., 2020). c: in backcross to *H. cydno*. m: in backcross to *H. melpomene*

Compound	Type	Mean (*H. melpomene*, ng)^a^	Mean (*H. cydno*, ng)^a^	Bonferroni‐significant QTL on chromosome	QTL position on chromosome (bp)	Percent parental difference explained by genotype at QTL position
(*Z*)‐9‐Octadecenal	Unsaturated aldehyde	10.94	Absent	1^m^	9955174^m^	33.544%^m^
Benzyl cyanide	Aromatic (nitrogenous)	Absent	1.813	17^m^	5596551^m^	67.126%^m^
Octadecanal^a^	Saturated aldehyde	783.4	0.569	20^c^; 20^m^	10947362^c^; 11725848^m^	0.602%^c^; 41.411%^m^
Henicosane	Alkane	12.45	1152	20^m^	12126129^m^	11.291%^m^
Methyloctadecanol	Saturated alcohol	12.23	Absent	20^m^	12374765^m^	43.435%^m^
1‐Octadecanol^a^	Saturated alcohol	345.6	Absent	20^m^	12652775^m^	27.709%^m^
(*Z*)‐16‐Methyl‐9‐octadecenol	Unsaturated alcohol	7.501	Absent	20^m^	12652775^m^	38.888%^m^
(*Z*)‐13‐Docosenal	Unsaturated aldehyde	62.56	0.276	20^c^	12917911^c^	28.582%^c^
(*Z*)‐11‐Icosenal	Unsaturated aldehyde	150.7	3.290	20^c^	13267611^c^	22.717%^c^
Methyl 3‐indoleacetate	Aromatic (nitrogenous)	3.782	0.013	Z^c^	8478732^c^	59.220%^c^

**TABLE 3:** Details of compounds with Bonferroni‐significant QTL in *Heliconius* genitals. a: from (Darragh et al., 2020). c: in backcross to *H. cydno*. m: in backcross to *H. melpomene*

Compound	Type	Mean (*H. melpomene*, ng)^a^	Mean (*H. cydno*, ng)	Bonferroni‐significant QTL on chromosome	QTL position on chromosome (bp)	Percent parental difference explained by genotype at QTL position
Hexyl 3‐methylbutyrate	Saturated ester	Absent	421.1	3^m^	234227^m^	5.863%^m^
2‐*sec*‐Butyl‐3‐methoxypyrazine	Aromatic (nitrogenous)	32.66	3.569	5^c^	4427722^c^	38.866%^c^
12‐Octadecanolide	Macrolide	Absent	105.4	8^c^	4512378^c^	31.434%^c^
(*Z*)‐9‐Octadecen‐11‐olide	Macrolide	Absent	7509	8^m^	5341821^m^	12.192%^m^
(*Z*9,*E*11)‐ 9,11‐Octadecadien ‐13‐olide	Macrolide	Absent	19714	8^m^	5341821^m^	2.826%^m^
Hexadecenolide	Macrolide	6.866	165.5	8^m^	5341821^m^	170.403%^m^
Hexyl octadecanoate	Saturated ester	121.8	24.21	8^m^	5987924^m^	46.335%^m^
(*Z*)‐9‐Octadecen‐13‐olide	Macrolide	7.282	5856	8^m^	6072562^m^	15.975%^m^
Isopropyl oleate	Unsaturated ester	50.22	2259	14^m^	7370836^m^	16.385%^m^

**TABLE 4:** Details of quantitative trait loci passing a significance threshold of either *p* = .05 or a Bonferroni‐corrected threshold

Compound (highlighted = Bonferroni‐significant QTL)	Compound type	Mapped in which backcross direction?	Chromosome	Linkage group	QTL peak position (cM)	QTL peak position (bp)	Bayesian confidence interval (cM)	Bayesian confidence interval (bp)	Percent parental difference explained by genotype at QTL position	Significance level
Wing compounds										
Henicosane	Alkane	to *H. melpomene*	20	Hmel220003o	49.55	12126129	49.55–56.37	12126129–14572192	11.291%	Bonferroni
Icosane	Alkane	to *H. melpomene*	20	Hmel220003o	53.72	12917911	48.03–56.37	11779327–14572192	14.531%	*p* = .05
Methyloctadecanol	Saturated alcohol	to *H. melpomene*	20	Hmel220003o	50.31	12374765	43.11–56.37	9369865–14572192	43.435%	Bonferroni
1‐Octadecanol	Saturated alcohol	to *H. melpomene*	20	Hmel220003o	51.82	12652775	10.91–56.37	1642405–14572192	27.709%	Bonferroni
Icosanal	Saturated aldehyde	to *H. cydno*	20	Hmel220003o	54.47	13171256	43.87–56.37	10168518–14572192	12.782%	*p* = .05
Octadecanal	Saturated aldehyde	to *H. melpomene*	20	Hmel220003o	47.66	11725848	46.90–56.37	11161989–14572192	41.411%	Bonferroni
Octadecanal	Saturated aldehyde	to *H. cydno*	20	Hmel220003o	45.76	10947362	42.35–54.85	9267450–13276882	0.602%	Bonferroni
(*Z*)‐11‐Icosenol	Unsaturated alcohol	to *H. cydno*	20	Hmel220003o	55.23	13279240	41.97–56.37	9257056–14572192	8.816%	*p* = .05
(*Z*)‐13‐Docosenol	Unsaturated alcohol	to *H. melpomene*	20	Hmel220003o	35.16	7515365	10.91–53.34	1642405–12908798	101.480%	*p* = .05
(*Z*)‐16‐Methyl‐9‐octadecenol	Unsaturated alcohol	to *H. melpomene*	20	Hmel220003o	51.82	12652775	46.90–56.37	11161989–14572192	38.888%	Bonferroni
(*Z*)‐11‐Icosenal	Unsaturated aldehyde	to *H. cydno*	20	Hmel220003o	54.85	13267611	45.76–56.37	10947362–14572192	22.717%	Bonferroni
(*Z*)‐13‐Docosenal	Unsaturated aldehyde	to *H. cydno*	20	Hmel220003o	53.72	12917911	45.00–56.37	10642774–14572192	28.582%	Bonferroni
(*Z*)‐9‐Octadecenal	Unsaturated aldehyde	to *H. melpomene*	1	Hmel201001o	33.92	9955174	30.13–39.60	8218856–12832776	33.544%	Bonferroni
(*Z*)‐9‐Octadecenal	Unsaturated aldehyde	to *H. melpomene*	20	Hmel220003o	55.23	13279240	41.60–56.37	9243035–14572192	30.508%	*p* = .05
Benzyl cyanide	Aromatic (nitrogenous)	to *H. melpomene*	10	Hmel210001o	48.87	14461296	0.00–56.87	9690–17959960	59.570%	*p* = .05
Benzyl cyanide	Aromatic (nitrogenous)	to *H. melpomene*	17	Hmel217001o	26.34	5596551	20.28–42.25	4078122–10433378	67.126%	Bonferroni
Benzyl cyanide	Aromatic (nitrogenous)	to *H. cydno*	19	Hmel219001o	3.34	606313	0.57–56.75	9879–16007669	130.336%	*p* = .05
Methyl 3‐indoleacetate	Aromatic (nitrogenous)	to *H. cydno*	21	Hmel221001o	28.8	8478732	0.00–53.43	5031–13351436	59.220%	Bonferroni
Genital compounds										
Docosane	Alkane	to *H. melpomene*	14	Hmel214004o	44.79	7697378	0.46–49.71	3905–8850536	45.983%	*p* = .05
Henicosane	Alkane	to *H. cydno*	20	Hmel220003o	51.06	12488808	47.66–56.37	11725848–14572192	43.720%	*p* = .05
Butyl hexadecanoate	Saturated ester	to *H. melpomene*	14	Hmel214004o	49.34	8723724	0.46–49.71	3905–8850536	37.817%	*p* = .05
Hexyl‐3‐methylbutyrate	Saturated ester	to *H. melpomene*	3	Hmel203003o	4.07	234227	0.48–12.4	4867–2439819	5.863%	Bonferroni
Hexyloctadecanoate (RI = 2590)	Saturated ester	to *H. melpomene*	8	Hmel208001o	30.87	5987924	0.93–48.72	41886–9309727	46.335%	Bonferroni
Octadecanoic acid ester	Saturated ester	to *H. cydno*	6	Hmel206001o	0.00	4240	0.00–4.54	4240–374231	81.615%	*p* = .05
Butyl oleate	Unsaturated ester	to *H. melpomene*	1	Hmel201001o	33.92	9955174	32.02–53.99	9550244–16234374	45.537%	*p* = .05
Butyl oleate	Unsaturated ester	to *H. melpomene*	2	Hmel202001o	25.82	4658742	0.80–53.9	10283–8996050	69.303%	*p* = .05
Butyl oleate	Unsaturated ester	to *H. cydno*	9	Hmel209001o	15.42	2427002	2.54–45.73	10536–8672501	3.995%	*p* = .05
Isopropyl oleate	Unsaturated ester	to *H. melpomene*	14	Hmel214004o	41.76	7370836	0.46–49.71	3905–8850536	16.385%	Bonferroni
2‐*sec*‐Butyl‐3‐methoxypyrazine	Aromatic (nitrogenous)	to *H. cydno*	5	Hmel205001o	24.39	4427722	0.91–51.7	9444–9821895	38.866%	Bonferroni
Benzyl cyanide	Aromatic (nitrogenous)	to *H. melpomene*	17	Hmel217001o	24.07	5377468	13.84–46.04	2863810–14762684	1.960%	*p* = .05
(*Z*)‐9‐Octadecen‐11‐olide	Macrolide	to *H. melpomene*	8	Hmel208001o	30.49	5341821	0.93–48.72	41886–9309727	12.192%	Bonferroni
(*Z*)‐9‐Octadecen‐13‐olide	Macrolide	to *H. melpomene*	8	Hmel208001o	31.25	6072562	0.93–48.72	41886–9309727	15.975%	Bonferroni
(*Z*9,*E*11)‐ 9,11‐Octadecadien −13‐olide	Macrolide	to *H. melpomene*	8	Hmel208001o	30.49	5341821	0.93–48.72	41886–9309727	2.826%	Bonferroni
12‐Octadecanolide	Macrolide	to *H. cydno*	8	Hmel208001o	24.81	4512378	0.93–48.72	41886–9309727	31.434%	Bonferroni
9‐Octadecen‐18‐olide	Macrolide	to *H. cydno*	20	Hmel220003o	3.33	237724	1.82–27.58	21924–6617001	50.049%	*p* = .05
Brassicalactone	Macrolide	to *H. cydno*	14	Hmel214001o	0.46	3905	0.46–49.71	3905–8850536	70.539%	*p* = .05
Icosen‐13‐olide	Macrolide	to *H. melpomene*	13	Hmel213001o	17.47	5860206	0.00–35.27	11191–13125309	7.936%	*p* = .05
Hexadecen‐11‐olide	Macrolide	to *H. cydno*	7	Hmel207001o	46.71	11245622	34.20–60.34	7556459–14241586	26.673%	*p* = .05
Hexadecenolide	Macrolide	to *H. melpomene*	8	Hmel208001o	30.49	5341821	0.93–48.72	41886–9309727	170.403%	Bonferroni
(*Z*9,*E*11,*Z*15)‐9,11,15‐ Octadecatrien‐13‐olide	Macrolide	to *H. melpomene*	2	Hmel202001o	14.84	2034014	0.80–53.9	10283–8996050	13.320%	*p* = .05
(*E*)‐β‐Ocimene	Terpenoid	to *H. cydno*	6	Hmel206001o	35.98	8298418	16.66–45.45	3143646–10034766	6.873%	*p* = .05

Compounds that showed significant QTL tended to be of specific chemical classes. For the wings, of the 14 wing compounds with significance at *p* = .05, 12 were fatty acid‐derived compounds (FADs), including alkanes as well as oxygenated alkanes and alkenes (aldehydes and alcohols). The remaining two significant wing compounds were both nitrogenous aromatics. For the genitals, 10 compounds producing significant QTL were macrolides and eight were FADs (two alkanes, four saturated esters, and two unsaturated esters). The remaining three compounds were again nitrogenous aromatics (two compounds) and one terpene. This is in general agreement with the distribution of compound types in both body regions. The mapped wing compounds comprise 18 fatty acid‐derived compounds, three nitrogenous aromatics, two non‐nitrogenous aromatics, and two terpenoids, so the finding of significant QTL mostly for fatty acid‐derived compounds is not surprising. For the genitals, the mapped compounds consist of 22 fatty acid‐derived compounds, 21 macrolides, four terpenoids, two nitrogenous aromatics, and one lactone.

**FIGURE 3:**
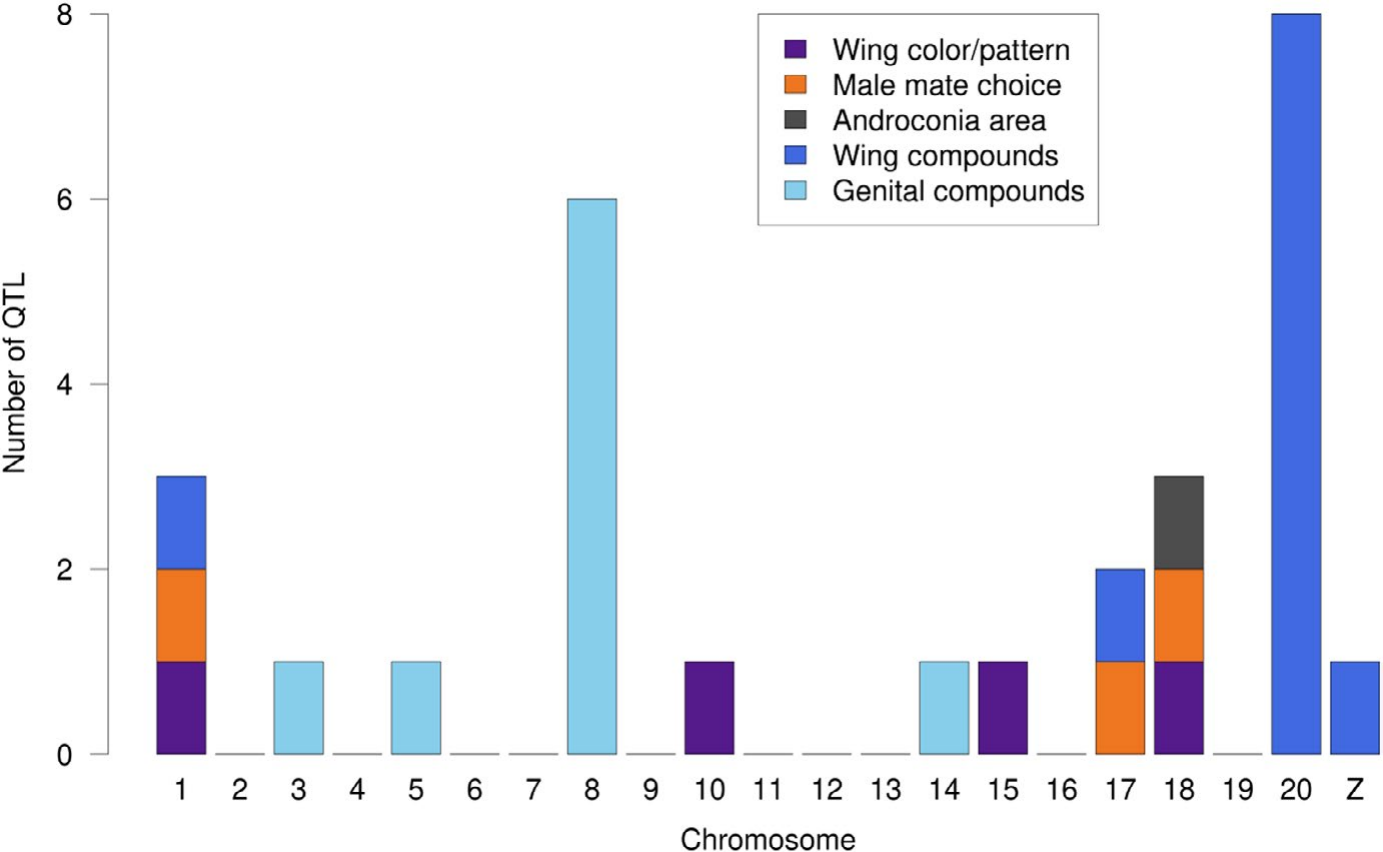
QTL for various traits across the*Heliconius melpomene*and*H. cydno*genomes. Wing color/pattern loci represent the four major color loci (Jiggins, 2017); male mate choice loci are from (Merrill et al., 2019); (relative) androconia area and Bonferroni‐significant wing and genital compound loci from this study.

**FIGURE 4:**
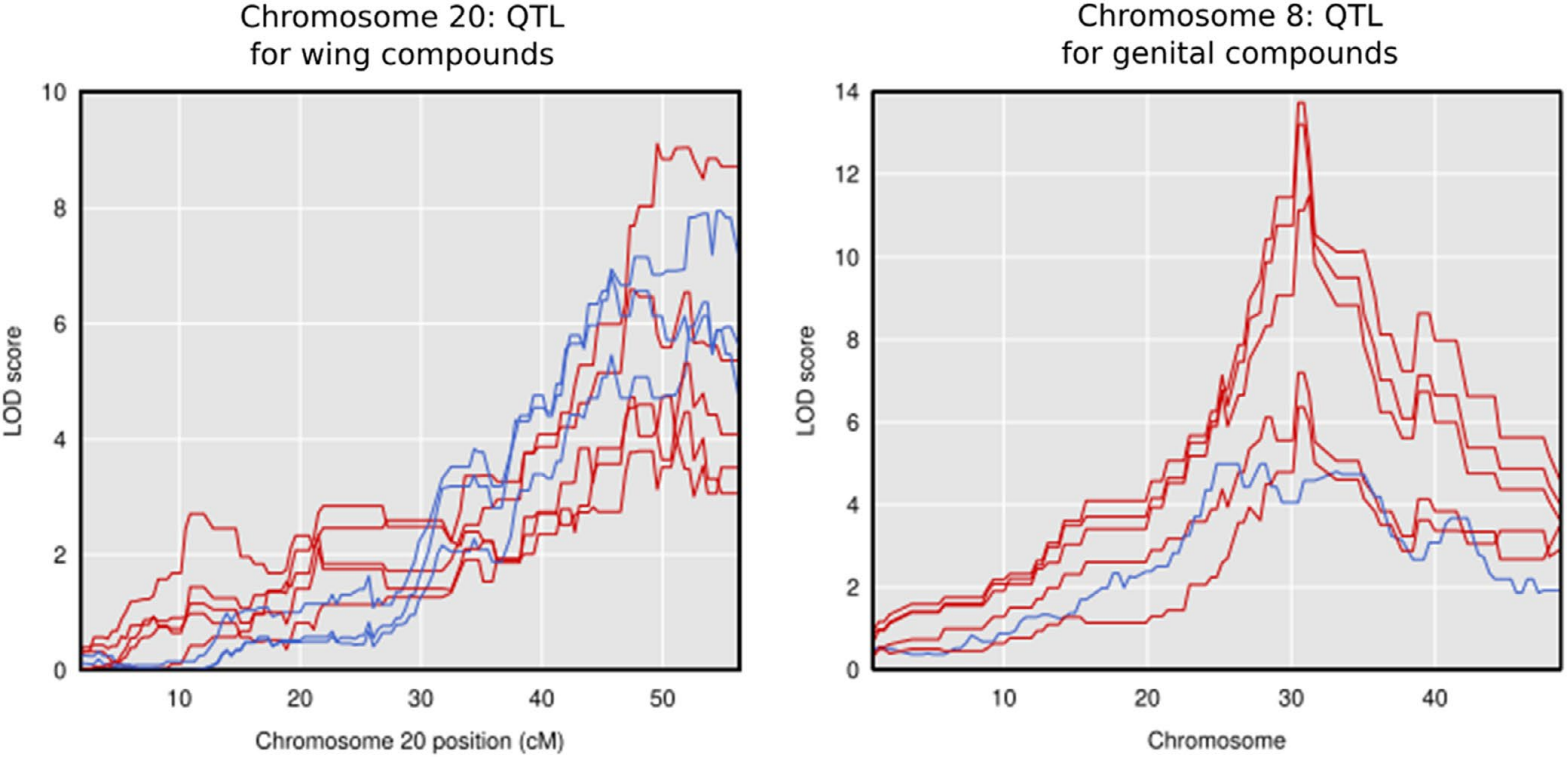
Chromosome‐specific QTL plots for compounds with significant QTL (after Bonferroni correction) on those chromosomes, with the entire chromosome length shown. Red: compound mapped in backcrosses to*H. melpomene*; blue: compound mapped in backcrosses to*H. cydno*

Significant quantitative trait loci showed some same‐chromosome clustering on specific chromosomes (Figure S4). Notably, these clusters did not overlap with chromosomes harboring known QTL for color pattern, mate choice, or other traits, which generally tend not to be clustered in the genome, apart from a close association between mate choice and a wing patterning gene (Figure 3). When either all QTL significant at *p* < 0.05 or Bonferroni‐corrected significant QTL were combined with known QTL, clustering at the chromosomal level was statistically significant (χ^2^ = 64.036 for Bonferroni‐significant QTL, χ^2^ = 78.524 for all significant QTL, *p* < .001 for both) for chromosomes 8 and 20. In particular, all but one QTL for FAD compounds in the wings mapped to chromosome 20 (the exception mapped to chromosome 1 as well as having a peak on chromosome 20). The other significant wing compounds (all nitrogenous aromatics) mapped to chromosomes 10, 17, 19, and the Z chromosome (chromosome 21). QTL on chromosome 20 were broadly overlapping, in part due to our relatively small mapping populations producing broad Bayesian confidence intervals for QTL location (Figure 4). In addition, the peaks themselves were strongly overlapping on the latter half of chromosome 20. This chromosome‐level clustering was statistically significant (χ^2^ = 178.39 for all significant QTL, χ^2^ = 113.02 for Bonferroni‐corrected significant QTL, *p* < .001 in both cases) and residual analysis identified chromosome 20 as the sole outlier. When broken down by chemical class, only FAD compounds clustered significantly at the chromosomal level, again on chromosome 20 (χ^2^ = 232.49 for all significant QTL, *p* < .001), and this was also significant when only Bonferroni‐corrected significant QTL were tested (χ^2^ = 137.55, *p* < .001).

Similar compound class‐specific clustering at the chromosome level was seen to a lesser extent for the genitals, where six of the ten macrolides had QTL on chromosome 8, with the others more broadly dispersed on chromosomes 2, 7, 13, 14, and 20. The fatty acid‐derived compounds (mostly esters) mapped more broadly across the genome than in the wings, with QTL on chromosomes 1, 2, 3, 6, 8, 9, 14, and 20. Chromosome‐level clustering was again statistically significant (χ^2^ = 46.037 for all significant QTL, χ^2^ = 101.83 for Bonferroni‐corrected significant QTL, *p* = .0017 and *p* < .001 respectively) and residual analysis identified chromosomes 8 and 14 as the outliers when all QTL were considered, and chromosome 8 when only Bonferroni‐corrected QTL were included. The clustering on chromosome 8 is solely due to the presence of macrolide QTL on this chromosome, while chromosome 14 contains QTL for both a macrolide and three FAD compounds. When broken down by chemical class, only macrolide compounds clustered significantly at the chromosomal level in the genitals, again on chromosome 8 (χ^2^ = 58.079 for all significant QTL, *p* < .001), and this was also significant when only Bonferroni‐corrected significant QTL were tested (χ^2^ = 110.88, *p* < .001). Again, the QTL peaks themselves were strongly overlapping in the middle of chromosome 8 (Figure 4) rather than being dispersed across the chromosome. Two compounds had significant QTL in both wings and genitals: henicosane and benzyl cyanide. Both data sets showed QTL on the same chromosomes (20 and 17, respectively, with additional QTL for benzyl cyanide in the wings on other chromosomes). The henicosane QTL were within 1.5 cM of one another, while the benzyl cyanide QTL on chromosome 17 were 2.3 cM apart.

**FIGURE 5:**
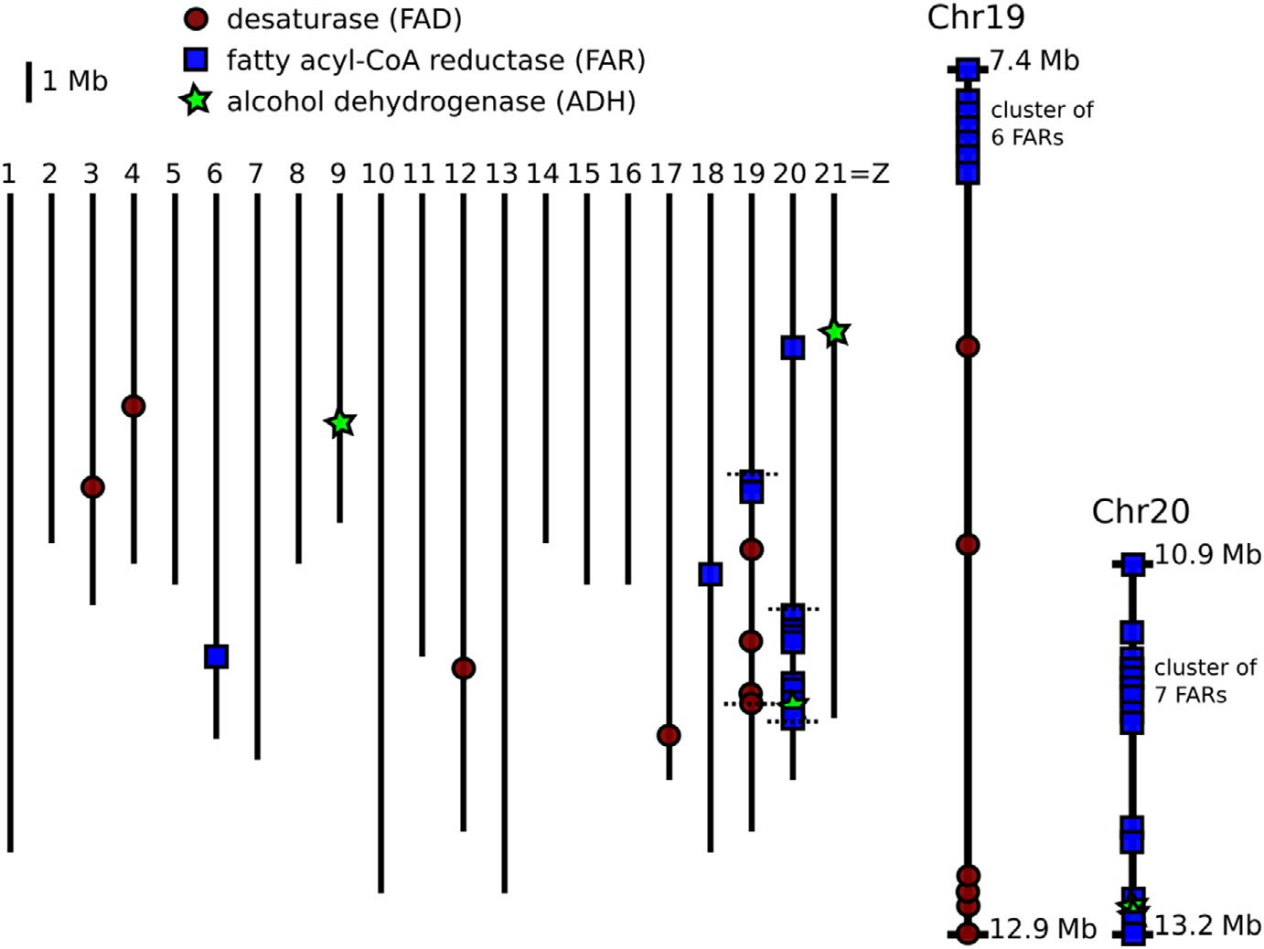
Location of candidate genes for fatty acid‐derived compound biosynthesis across the genome of*Heliconius melpomene*. Horizontal dotted lines on chromosomes 19 and 20 indicate the endpoints of zoomed in regions

The strong clustering of QTL for wing fatty acid‐derived compounds on chromosome 20 suggested a common gene or set of linked genes responsible for fatty acid‐derived compound metabolism or its regulation. We searched the region between 43.11 and 56.37 cM (9,398,348–14,585,564 basepairs) for potential candidate genes that could be responsible for this clustering. This region spanned the confidence intervals for all Bonferroni‐significant QTL apart from 1‐octadecanol and contained 363 genes. Annotated proteins from this region (from Lepbase, Challis et al., 2016) were searched using BLASTp against the NCBI nonredundant (nr) database, revealing a total of 20 genes potentially involved in the biosynthesis of the wing compounds within this confidence interval on chromosome 20. These genes include two acetyl‐CoA carboxylases (involved in primary metabolism), fifteen fatty acyl‐CoA reductases (FARs), and two alcohol dehydrogenases (the latter two families involved in both primary and secondary metabolism), none of which have been functionally characterized. Comparing our candidates to those identified in Liénard et al. (2014), none of our FARs fall within the pheromone gland FAR (pgFAR) clade, whose members fall on chromosome 19. Inspecting the genome for additional secondary metabolism fatty acid‐derived metabolism genes, we identified clusters of FARs on chromosomes 19 and 20 (Figure 5), with a more even distribution of alcohol dehydrogenases and desaturases across the genome. Notably, we did not identify any QTL on chromosome 19 for fatty acid‐derived compound production in the wings.

## 4 DISCUSSION

The genetic basis for traits contributing to reproductive isolation remains poorly studied. This is especially the case for differences in chemical signaling between closely related nonmodel species, in particular for species with complex chemical signatures where multiple components may play a role in mate choice or species recognition. Here, we mapped a total of 40 QTL controlling the segregation of wing and genital pheromone bouquet compounds in crosses between *Heliconius melpomene* and its close relative *H. cydno*. There was significant evidence for nonrandom clustering of QTL, most notably for wing compounds on chromosome 20, which showed broadly overlapping QTL with closely spaced peaks. There could be a number of reasons for this clustering of loci. First, theory predicts that mutations controlling species differences may accumulate, and persist, in regions already resistant to gene flow, such that recombination is less likely to disrupt species differences (Smadja & Butlin, 2011). Second, functionally similar genes are likely to occur in close linkage due to, for example, tandem gene duplication, such that chemically similar compounds could be regulated by linked genes due to functional similarities between their synthesis pathways (Osbourn, 2010). Finally, related compounds could differ between species as a pleiotropic effect of the same mutation if they are downstream products of the same biochemical pathway or in the case of a multifunctional enzyme. The end result of all of these processes is linkage that can facilitate speciation by impeding recombination that will otherwise disrupt the genetic associations between traits that characterize emerging species.

In the case of the compounds described here, it is likely that shared biosynthetic origins play an important role in their chromosomal distributions. The majority of the wing compounds found in either species are fatty acid‐derived compounds (FADs; 19 of 31), and the majority of these FADs (12 of 19) were associated with a significant QTL on chromosome 20. It is interesting that there was clustering of one class of biosynthetic enzyme in this pathway, the fatty acyl‐CoA reductases (FARs), on both chromosomes 19 and 20, but no QTL on chromosome 19 for any wing or genital FAD compound, suggesting the FARs on chromosome 19 are not involved in putative pheromone compound production. Similarly, QTL for genital compounds exhibited slight clustering of loci underlying macrolide compound production on chromosome 8, but otherwise were more evenly spread across the genome, likely due to their greater biosynthetic diversity. The QTL for shared macrolide compounds may also be due to shared biosynthetic origins of these compounds, but the biosynthesis of macrolides is less well understood and consequently we were unable to identify candidate genes on this chromosome. We also found a QTL on chromosome 3 for hexyl 3‐methylbutyrate, a known bioactive component of the genital pheromone of *H. cydno* (Estrada, 2009) whose genetic basis was previously unknown. Of note, the QTL for relative androconial area roughly peaks on the gene *optix* (chromosome 18), a locus‐controlling red color patterns in *Heliconius* (Reed et al., 2011; Van Belleghem et al., 2017). This locus is also known to control male‐specific scales in the basal heliconiine *Dryas iuila* and has an expression domain in the hindwing androconia region (Martin et al., 2014). This overlap raises the possibility for coupled control of color pattern and androconial pheromone production differences between *Heliconius* species.

Overall, we found no evidence for linkage of any of the chemical QTL clusters with other loci or QTL that have been shown to play a difference in the strong assortative mating observed between *H. melpomene* and *H. cydno*. The major wing patterning genes are located on chromosomes 1 (*aristalless*), 10 (*WntA*), 15 (*Cortex*), and 18 (*optix*), only chromosome 1 of which harbors a chemical QTL, but the peak does not include the *aristalless* gene. In addition, major QTL for male mate choice map to chromosome 1, 17, and 18 (Merrill et al., 2019), but, similarly, the chemical QTL we identified on chromosomes 1 and 17 do not overlap with the major QTL identified for male mate choice. We might expect an overlap between chemical QTL and female mate choice QTL (as females likely assess males partially based on their wing androconial chemistry), but the latter has not been mapped in this species pair. Nonetheless, the clustering of wing compounds in particular might contribute to pheromone‐based speciation, particularly in the face of gene flow (Via, 2012). If more than one pheromone component that maps to chromosome 20 is involved in reproductive isolation, and if genes responsible for their production are tightly linked (or if pleiotropy occurs), this may also facilitate speciation (Felsenstein, 1981; Merrill et al., 2019; Smadja & Butlin, 2011) and prevent the breakup of potentially adaptive pheromone bouquet mixtures. Evidence suggests that mixture effects are important in pheromone processing (Clifford & Riffell, 2013; Lei et al., 2013; Riffell et al., 2009), and these mixtures might be maintained through tight genetic linkage between biosynthetic loci, facilitated by gene evolutionary events such as tandem duplication. In contrast to the tight clustering seen on chromosome 20 for wing FAD compounds, we saw more limited clustering for the more chemically diverse genital compounds, likely due to their more diverse potential biosynthesis pathways. This might hamper any potential role in reproductive isolation. Given their known function as antiaphrodisiacs, genital compounds are not so likely to play a major role in isolation between *Heliconius* species (Estrada, 2009; Gilbert, 1976; Schulz et al., 2007).

Some of the compounds found on wings and in genitalia might have a plant origin. Several compounds in the wings and genitals did not differ between parental species, and many of these have been shown to be affected by larval diet in *Heliconius melpomene*. Most of the invariant wing compounds are aromatics, which are expected to be plant derived in *Heliconius* (Darragh, et al., 2019a). Notably, we reared all mapping butterflies on the same larval and adult diets, thus effectively creating a dietary common garden experiment, and so we do not expect larval or adult diet to affect the result of the QTL mapping. Many male moth pheromone components are derived from host plant volatiles with minimal alterations, potentially due to females’ pre‐existing biases for detecting these compounds during the host plant search (Conner & Iyengar, 2016). However, many of our mapped *Heliconius* compounds do appear to have a genetic basis (i.e., a QTL was found), in line with the biosynthesis of FAD and related compounds by female moths. Breaking our compounds down into four classes (FADs, macrolides, aromatics, and terpenoids), we failed to find QTL (at the *p* = .05 level) for 54%, 58%, 43%, and 83% of these classes, respectively, across both wings and genitals. In particular, terpenoids that differ between species may be the result of differential larval host plant use. It is of course possible that plant derived compounds could show a genetic basis if there were species differences in the genes regulating their uptake, and one example might be icosanal, which has been shown to be influenced by larval host plant (Darragh, et al., 2019a) but which is also associated with a QTL in this study.

This study provides a first step toward better characterizing the genes causing chemical differences between species. Fatty acid‐derived compound biosynthesis candidate genes and QTL for FAD compounds are both clustered on chromosome 20. These FAD compounds are produced from acetyl‐CoA followed by rounds of chain elongation, desaturation, fatty acyl‐CoA reduction, oxidization, and acetyltransferase activity to produce the final pheromone compounds. The fatty acyl‐CoA reduction step, key to pheromone biosynthesis, uses FARs to remove the CoA moiety and return an alcohol. More than half of these FAR candidates are found in tandem duplications, as are the two alcohol dehydrogenase candidates. These duplications could have provided raw material for the origin of new biosynthetic function and thus increased pheromone diversity. FARs also cluster on chromosome 19, and these FARs (unlike those on chromosome 20) fall within the pheromone gland FAR (pgFAR) subfamily (Liénard et al., 2014; Löfstedt et al., 2016). However, these phylogenetic analyses of FARs in *Heliconius*, *Bombyx, Danaus,* and *Bicyclus* do not generally show lineage‐specific clustering of the chromosome 20 FARs; instead, they are dispersed throughout the tree. This suggests an ancient evolutionary origin of the *Heliconius* chromosome 20 FAR cluster, in contrast to *Heliconius*‐specific duplications of chromosome 19 FARs in the pgFAR subfamily which are clustered in the tree.

Single FAR and alcohol dehydrogenase genes are missing from *H. cydno* (Byers et al., 2020), suggesting an obvious mechanism for the striking difference in FAD compounds between the two species; these differences may also be due to, for example, differential regulation of FARs or other biosynthetic genes, or mutations within these genes. Alternately, a single FAR gene or small set of genes may be responsible for the biosynthesis of the majority of FAD compounds in *Heliconius*, similar to the situation found with *eloF* in *Drosophila*, where knockdown of the gene produces widely pleiotropic effects on cuticular hydrocarbon profiles (Combs et al., 2018). Similar effects are seen in *Spodoptera litura*, where RNAi of *FAR17* alone alters levels of the four major sex pheromone components (Lin et al., 2017). Another option, though one we consider unlikely, is that this tight cluster of FARs on chromosome 20 serves as a supergene, allowing multiple phenotypic traits (here individual chemical compounds) to cosegregate and maintaining strong integration of the chemical phenotype. Supergenes have been described for other phenotypic morphs in *Heliconius* (Joron et al., 2011), but we are unaware of mechanisms on chromosome 20 that might contribute to supergene formation and maintenance, for example a chromosomal inversion, which would have likely been evident in our linkage map.

Information on potential biosynthetic gene clustering in other Lepidoptera species is largely lacking, as most studies have produced transcriptomes of pheromone glands rather than linkage maps or transcriptomes mapped to genomes. Additionally, as much work in Lepidoptera has focused on single pheromone components and thus single biosynthetic genes (Groot et al., 2016), clustering of QTL and candidate genes is rarely discussed. The lack of annotated genomes in many lepidopteran species also can prevent in‐depth assessment of clustering of candidate genes, requiring mapping QTL linkage groups back to *Bombyx* or other well‐annotated genomes. However, such clustering has been seen in *Nasonia* wasps, where both QTL for multiple cuticular hydrocarbon components and candidate desaturase genes overlap strongly across the genome (Niehuis et al., 2011, 2013). Clusters of acetyltransferases and desaturases exist in *Heliothis* (inferred from synteny with *Bombyx mori*), but the acetyltransferases do not appear to underlie the QTL for sex pheromone differences between *H. virescens* and *H. subflexa* (Groot et al., 2013, 2014). Quantitative trait loci for multiple compounds were investigated in *Heliothis*, finding multiple chromosomes responsible for the production of the same compound classes (all FADs), with more limited clustering (Sheck et al., 2006). This is similar in pattern to what we found in the genital pheromone components, although the latter cover multiple compound classes.

Understanding the genetic architecture of complex traits can allow us to understand their potential role in speciation. Determining whether the observed clustering QTL on chromosomes 8 and 20 reflect tight linkage of multiple biosynthetic and/or regulatory genes or a single “master” locus responsible for the biosynthesis or regulation of multiple pheromone components will contribute to our understanding of both pheromone biosynthesis and pleiotropy and linkage in general. With genes in hand, CRISPR (a technique that works in *Heliconius*, Livraghi et al., 2018) could be used to alter gene function, allowing confirmation of the role of pheromone components via behavioral assays. As major effect loci (as these QTL appear to be) and tight linkage (as potentially seen here) can both facilitate the speciation process, especially in the face of gene flow (Felsenstein, 1981; Merrill et al., 2010; Smadja & Butlin, 2011; Via, 2012), the QTL identified here could be barrier or speciation genes in *Heliconius*. These specific QTL may be involved in interspecific mate choice and reproductive isolation, and thus, the genes underlying them could contribute to the maintenance of species boundaries. We have here identified a total of 40 QTL underlying species differences in 33 potential pheromone components, demonstrated clustering of these QTL for wing and genital compounds on specific chromosomes not linked to known loci for species differences, and identified candidate genes underlying the production of the major chemical class of wing compounds. Together, these findings further our knowledge of chemical ecology, pheromone genetics, and their potential roles in *Heliconius* butterfly mate choice and speciation.

## CONFLICT OF INTEREST

The authors declare no competing interests.

## AUTHOR CONTRIBUTIONS

KJRPB and KD: Joint first author. CDJ, KD, and KJRPB: Study conception and design. KJRPB and KD: Phenotyping and QTL mapping for wing and genital compounds, respectively. KJRPB: Downstream analyses. KD and RMM: Design and QTL mapping crosses; KD and IAW: Sequencing libraries. KD, SFG, DAA, KJRPB, and RMM: Butterflies for mapping experiments. SS: Mass spectrometry libraries and advice on compound identification. PMAR: Raw sequencing data analysis, producing phased sequence data, and linkage map construction. CDJ and WOM: Funding and resources and, with RMM, QTL mapping experiment design. KJRPB: Manuscript writing; All authors: Manuscript editing. All authors: Final version of the manuscript.

## Supporting information

Figures S1‐S4

Figure S1

Figure S2‐part1

Fig S2‐part2

Figure S3

Figure S4

Tables S1 and S2

## Data Availability

Data and QTL analysis code have been deposited in Dryad under accessions https://doi.org/10.5061/dryad.rxwdbrv6j and https://doi.org/10.5061/dryad.crjdfn31b. Sequencing reads leading to linkage map construction have been deposited in the European Nucleotide Archive (ENA) under project PRJEB34160.
