# Supplementary material for: Clustering of loci controlling species differences in male chemical bouquets of sympatric Heliconius butterflies
Source: Ecol Evol. 2020 Dec 16;11(1):89–107. doi: 10.1002/ece3.6947 (PMC7790645; doi:10.1002/ece3.6947)

**Appendix: Supplemental figures**

Supplemental Figure 1: QTL plots for all mapped wing compounds. Dashed horizontal line: p = 0.05 significance threshold. Solid horizontal line: Bonferroni-corrected significance threshold. BC, backcross.


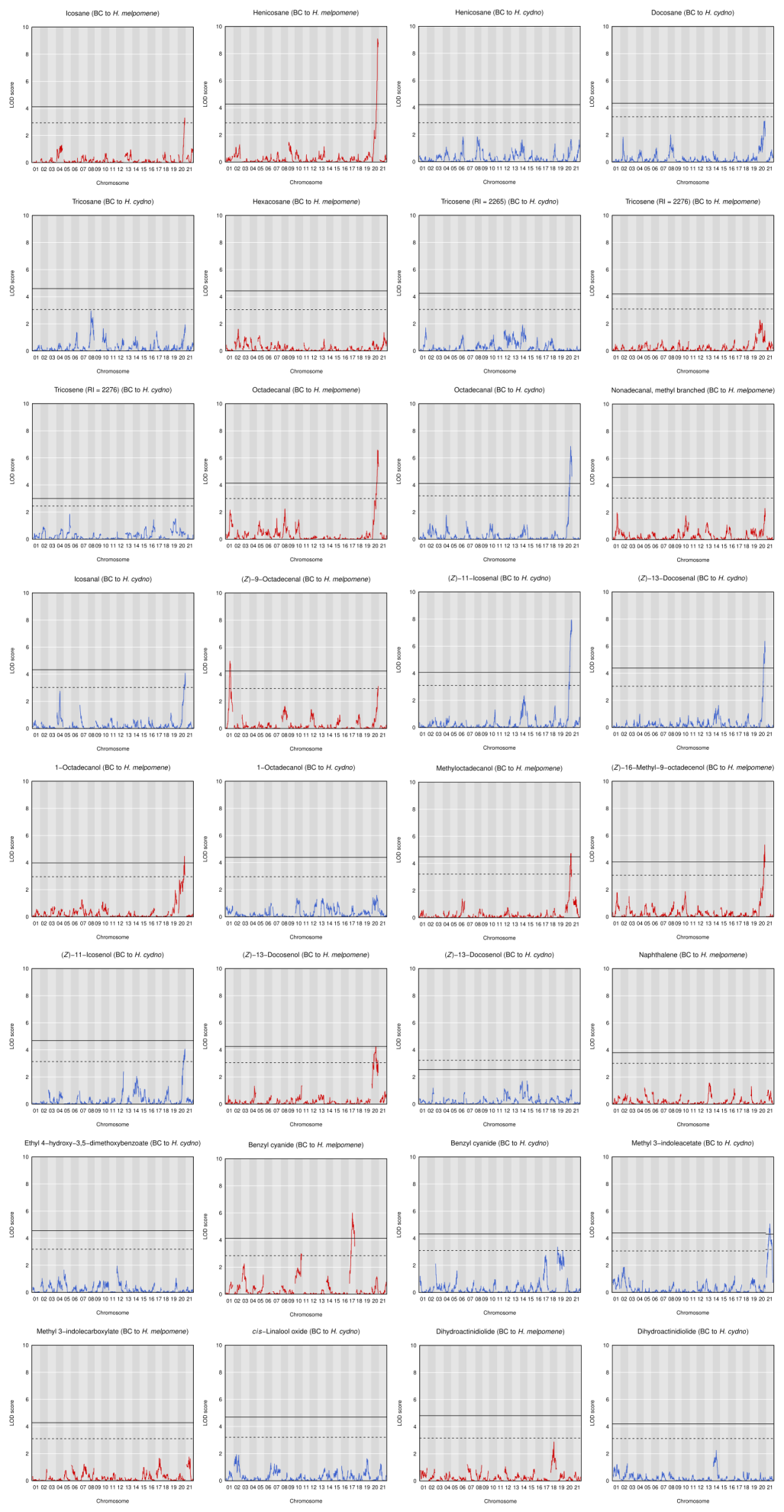


Supplemental Figure 2 (in two parts): QTL plots for all mapped genital compounds. Dashed horizontal line: p = 0.05 significance threshold. Solid horizontal line: Bonferroni-corrected significance threshold. BC, backcross.


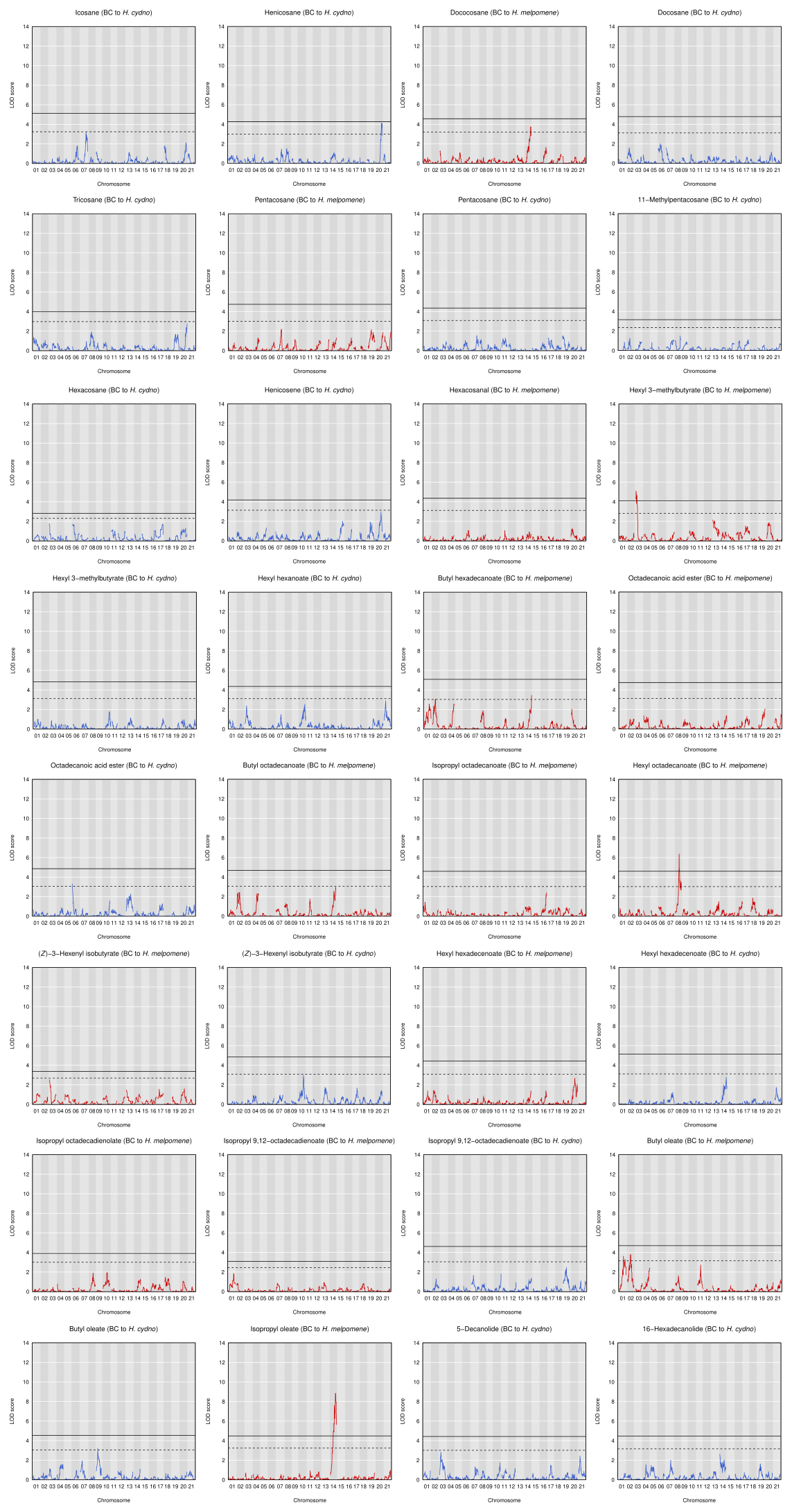

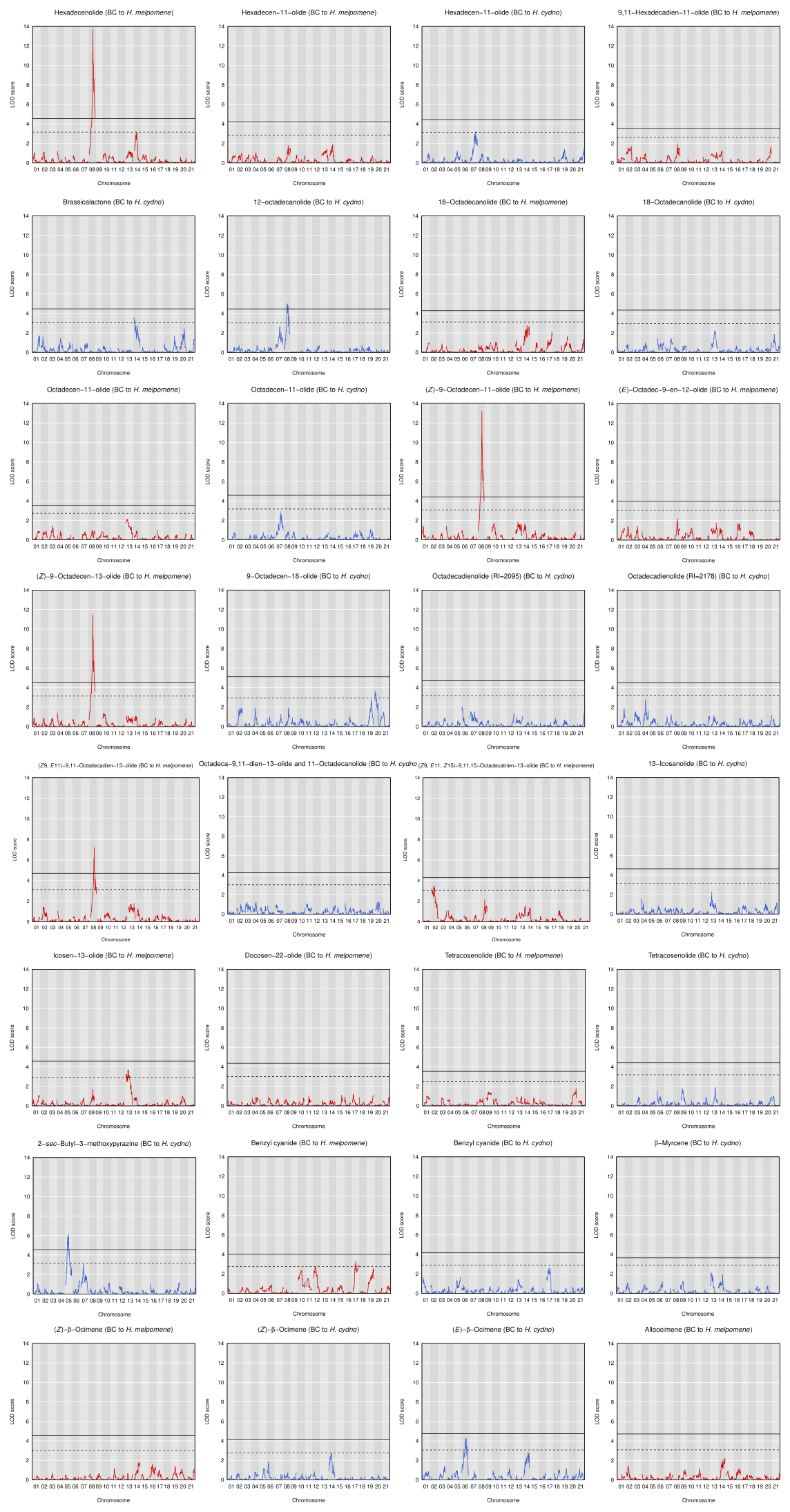


Supplemental Figure 3: QTL plots for hindwing androconial proportion. Solid horizontal line: p = 0.05 significance threshold. HW, hindwing. BC, backcross.


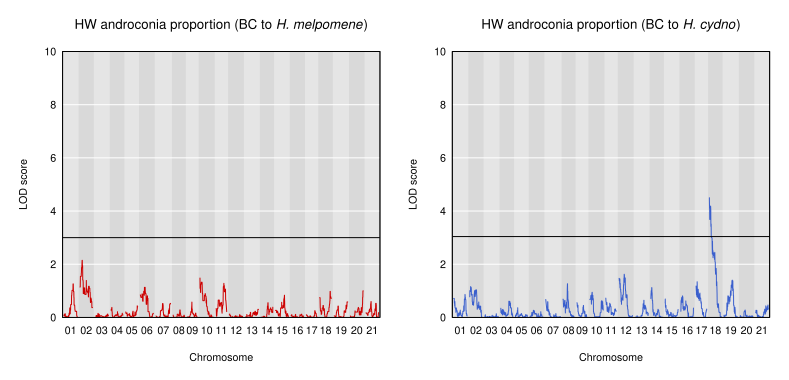


Supplemental Figure 4: Whole-genome QTL plots for compounds with at least one significant QTL. Red: compound mapped in backcrosses to *H. melpomene*; blue: compound mapped in backcrosses to *H. cydno*. A: Compounds with at least one QTL significant at the p = 0.05 level shown. B: Compounds with at least one QTL significant after Bonferroni correction shown.


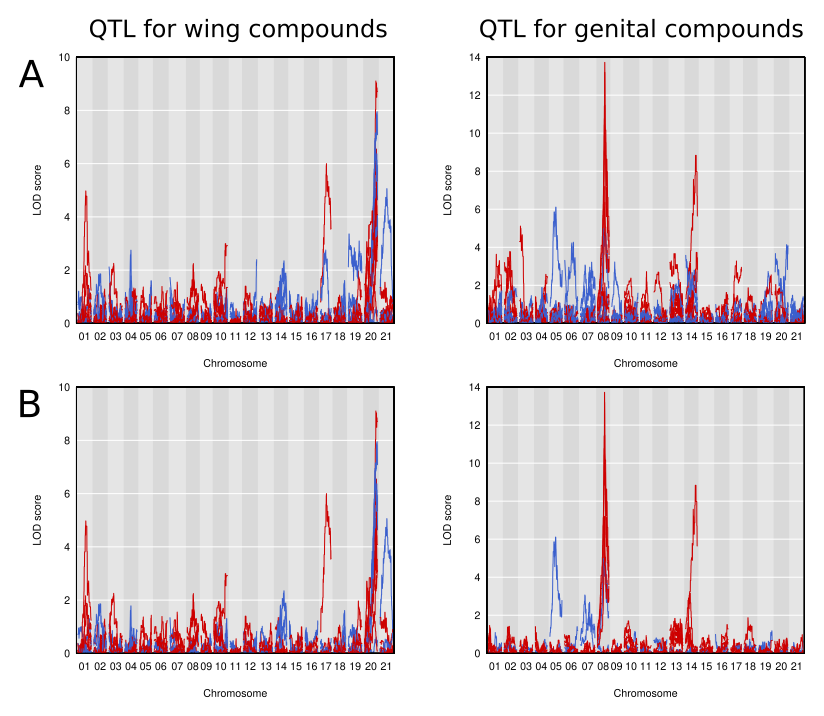

Supplement: Supplementary file 1 — Figures S1‐S4 [file ECE3-11-89-s001.docx]
